# Influence of pathological tumour variables on long-term survival in resectable gastric cancer

**DOI:** 10.1038/sj.bjc.6600161

**Published:** 2002-03-04

**Authors:** A Cuschieri, I C Talbot, S Weeden

**Affiliations:** Department of Surgery and Molecular Oncology, Ninewells Hospital and Medical School, University of Dundee, Dundee DD1 9SY, UK; Colorectal Cancer Unit, Imperial Cancer Research Fund and Academic Department of Pathology, St Mark's Hospital, Harrow HA1 3UJ, UK; Cancer Division, MRC Clinical Trials Unit, 222 Euston Road, London NW1 2DA, UK

**Keywords:** gastric cancer, pathology, tumour infiltrating eosinophils, prognostic factors, survival

## Abstract

Although tumour stage and nodal status are established prognostic factors for resectable gastric cancer, the relative importance of other pathological characteristics remains unclear. This study reports univariate and multivariate analyses of the prognostic value of various pathological and staging factors based on 324 patients entered into the MRC randomised surgical trial for gastric cancer. In the univariate analysis tumour stage, nodal status, UICC clinical stage, number of involved nodes, WHO predominant type, mixed Lauren type, Ming type, tumour differentiation, lymphocytic and tumour stromal eosinophilic infiltration were all found to have a significant impact on survival (logrank test, 5% level). In the multivariate analysis, UICC clinical stage and eosinophilic infiltration were found to have a significant influence. Risk of death increased for UICC stage II and III patients (Hazard Ratio for stage II compared to stage I=2.0, 95% Confidence Interval (CI) 1.4–2.9; Hazard Ratio for stage III compared to stage I=3.5, 95% CI 2.5–4.8). Patients with numerous eosinophils had a lower risk of death than those with none (Hazard Ratio=0.5, 95% CI 0.3–0.8). This association between survival and eosinophilic infiltration merits further study.

*British Journal of Cancer* (2002) **86**, 674–679. DOI: 10.1038/sj/bjc/6600161 www.bjcancer.com

© 2002 Cancer Research UK

## 

Carcinoma of the stomach is a major cause of death within the United Kingdom. The only proven effective therapy remains surgical resection though overall 5-year survival rates remain poor. In the 1980s, results from Japan suggested that gastrectomy with radical lymphadenectomy (D_2_ resection) improved survival over the standard D_1_ resection (Maruyama *et al*, 1987). Some Western centres practised and reported favourably on D_2_ resections (Sue-Ling *et al*, 1993) but the superiority of these operations was not tested prospectively until the launch of the Medical Research Council Gastric Cancer Surgical Trial (ST01) in 1986. In this prospective randomized study, D_1_ resection (removal of regional perigastric nodes) was compared with D_2_ resection (extended lymphadenectomy to include level 1 and 2 regional nodes). Central randomization followed staging laparotomy. Of 737 patients with histologically proven gastric adenocarcinoma registered, 337 were ineligible at staging laparotomy because of advanced disease and 400 were randomised.

The preliminary results of ST01 (Cuschieri *et al*, 1996) and a similar Dutch trial (Bonenkamp *et al*, 1995) had documented higher post-operative mortality and morbidity for patients randomised to D_2_ resection. This was thought to be a consequence of distal pancreatectomy and splenectomy, which were an integral part of most D_2_ procedures when these trials were designed. Long-term results of both trials have since failed to show a significant survival benefit to D_2_ surgery (Bonenkamp *et al*, 1999; Cuschieri *et al*, 1999).

An important aspect of ST01 was a full examination of all resected tumours by the pathology review panel. As well as determining tumour stage and nodal status, tumours were assessed using the WHO, Lauren, Mulligan and Ming classifications, in addition to grading based on the degree of differentiation. Extent of infiltration of the tumour stroma by lymphocytes and eosinophils was also assessed as some studies had suggested a potential survival benefit for patients with marked stromal infiltrates (Yu *et al*, 1995; Songun *et al*, 1996). The effect of these pathological and staging criteria on patient survival is examined in this study.

## MATERIALS AND METHODS

### Patients

Patients enrolled in MRC ST01 were to have had histologically proven and potentially curable gastric carcinoma. They were excluded if they were young (<20 years), had undergone previous gastric surgery, harboured a coexisting cancer or had serious co-morbid cardiorespiratory disease that precluded a safe D_2_ resection. All patients underwent staging laparotomy to define potentially curative disease. Eligible cases were those that fell within the Japanese gastric cancer stages I–III except those with positive infracolic aortic nodes. Within the same operating session patients were randomized centrally to receive either D_1_ or D_2_ surgery.

### Pathology

Pathologists at each local centre provided information on the size, differentiation and extent of the tumour, and on nodal groups. In addition a panel of specialist gastrointestinal pathologists reviewed the tumours. In this analysis, all staging criteria were defined using UICC classifications (Sobin and Wittekind, 1997). Tumour stage was assessed at pathology review. Nodal status and number of involved nodes were determined using information on nodal examination undertaken by local pathologists. These measurements were combined to give an overall clinical stage.

The review pathologists graded the tumours as well, moderately or poorly differentiated, and also assessed them using the WHO, Lauren, Mulligan and Ming classifications (Mulligan and Rember, 1954; Lauren, 1965; Ming, 1977; Watanabe *et al*, 1990). Eosinophilic and lymphocytic infiltration in the stroma of the tumour were also determined. The staining used for this purpose was a standard H&E and the microscope used for the study was a Zeiss Axioplan. A ‘high power field’ on this microscope (i.e., using a ×40 objective) measures 0.6 mm in diameter on the slide, giving an area of 1.88 mm^2^. The number of stromal eosinophils was recorded as ‘numerous’ (an average of five or more eosinophils in 10 such high power microscopic fields (HPFs)), ‘scanty’ (an average of less than five eosinophils in 10 HPFs) or ‘absent’ (no eosinophils present). Stromal lymphocytic infiltration was graded as ‘unremarkable’ or ‘heavy infiltrate’.

In total, 400 eligible patients were randomised into this trial. Local pathology data was collected for 386 patients. Due to logistical difficulties, only one reference pathologist (IC Talbot) was able to review the majority of the tumour slides (the other three pathologists were able to review only 8, 24 and 48% of the material). Using Talbot's assessments (carried out blind of the clinical outcome), complete prognostic information was available for 324 patients (154 D_1_, 170 D_2_). These form the basis of the present analyses.

Patients were followed up at regular intervals. For the patients within this analysis, follow-up is available to death or 3 years in 98% of patients and the median follow-up time is 8 years. Patients were followed up through the participating clinician, their GP or via the Office for National Statistics.

### Statistical methods

The statistical analysis was conducted using the SPSS software system. The primary endpoint of this analysis is survival, calculated from date of surgery. The univariate survival analyses were performed using the Kaplan–Meier method, and treatment comparisons were made via the log-rank test. Cox's proportional hazards technique was used to fit the multivariate survival model, significant prognostic factors were chosen using a forward conditional stepwise method. Categorical variables were fitted using dummy variables in the multivariate model, for ordinal variables the lowest value was used for reference. A significance level of 5% was adopted for all analyses. No significant difference in survival between D_1_ and D_2_ surgery was found for this trial so it was considered reasonable to combine the treatment arms for the purposes of this analysis.

## RESULTS

### Patient characteristics

The main characteristics of the 324 patients included in this analysis are shown in Table 1

**Table 1:**
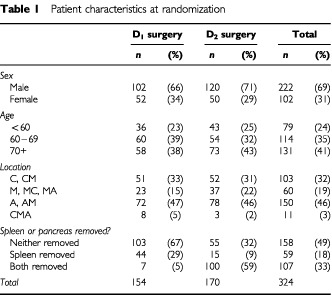
Patient characteristics at randomization

. Two-thirds of the patients are male, 40% were over 70 years old and nearly half had an antral tumour. The protocol advocated that patients in the D_2_ arm (excepting antral tumours) should receive a distal pancreatectomy and splenectomy, which explains the imbalance in this variable. The pathological characteristics recorded by the reference pathologist, and information on staging, are displayed in Table 2

**Table 2:**
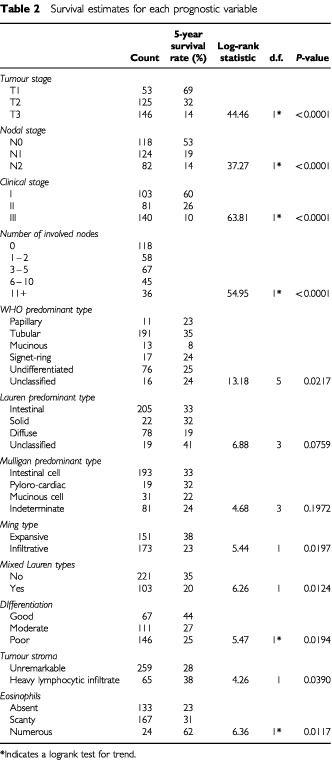
Survival estimates for each prognostic variable

.

### Univariate survival analysis

Table 2 contains counts and 5-year survival estimates for each level of each variable of interest. It can be seen that only Lauren predominant type and Mulligan predominant type fail to achieve significance at the 5% level. In order to establish the combined importance of the effects of these variables, a multivariate approach was used.

### Multivariate survival analysis

The results of the model fitting procedure can be seen in Table 3

**Table 3:**
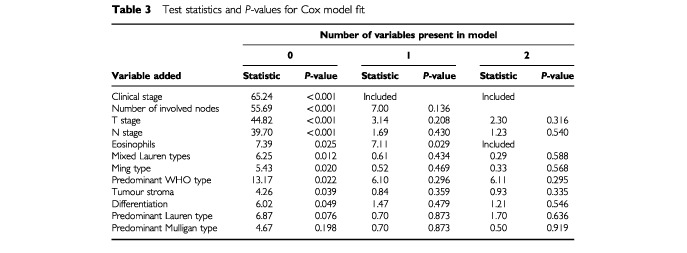
Test statistics and *P*-values for Cox model fit

**Figure 1:**
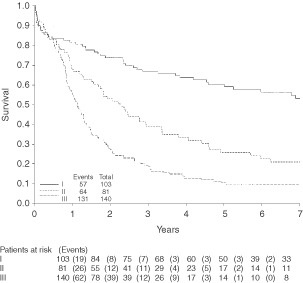
Survival by clinical staging.

. As in the univariate analysis, all of the variables except predominant Lauren type and predominant Mulligan type were significant when added into the initial model. Again clinical stage is the most important prognostic factor. When clinical stage was adjusted for, only extent of eosinophilic infiltration had a significant independent effect. Survival curves by clinical stage and eosinophil level are shown in Figures 1

and 2

**Figure 2:**
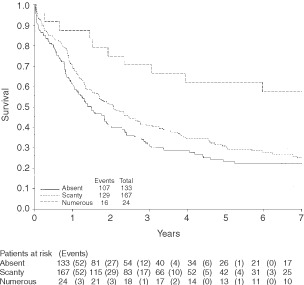
Survival by eosinophil level.

.

Hazard ratios, with 95% confidence intervals, for the significant variables are tabulated in Table 4

**Table 4:**
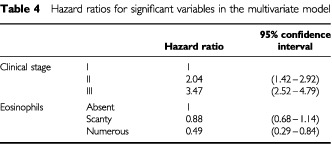
Hazard ratios for significant variables in the multivariate model

**Figure 3:**
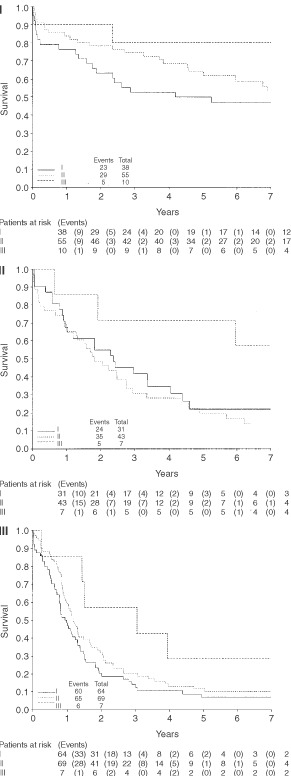
Survival by eosinophil level (stages I–III).

. Stage II patients have double the risk of death as stage I patients, and for stage III patients the risk is increased to 3.5 times that for stage I. A high level of eosinophils was associated with less than half the risk of death of those who have no eosinophils, however it should be noted that the group of patients with a high level of eosinophils is very small. The association between improved survival and a high eosinophil count is repeated for all stages, as can be seen in Figure 3

.

The relationship between stromal lymphocytic and eosinophilic infiltration is shown in Table 5

**Table 5:**
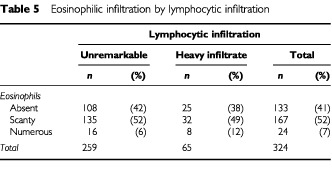
Eosinophilic infiltration by lymphocytic infiltration

. There was no significant correlation between these variables (χ^2^ statistic=2.89, *P*=0.24 on 2 d.f.).

Table 6

**Table 6:**
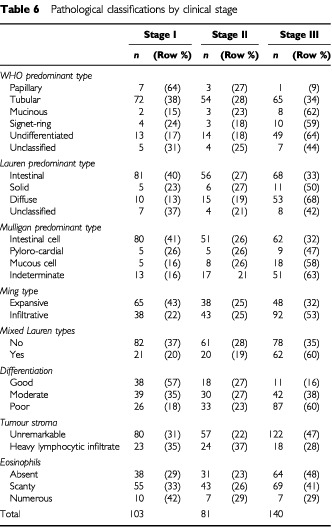
Pathological classifications by clinical stage

shows the pathological variables divided by clinical stage. This table gives an indication of why the significant effect of these variables detected on univariate analysis was not found in the multivariate analysis once clinical stage had been added into the model. For example, Table 2 suggests that patients with expansive Ming type have better survival than those with infiltrative type. It can be seen in Table 6 that 32% of expansive patients are stage III whereas 53% of infiltrative patients are stage III, thus ensuring lower survival for infiltrative patients. This pattern is repeated for many of the pathological variables.

## DISCUSSION

A detailed review published in 1995 (Hermanek *et al*, 1995) concluded that all large multivariate studies in gastric cancer find tumour stage and nodal status to have a significant prognostic influence, but the role of other variables is less clear. Apparent differences in prognosis for different WHO or Lauren sub-types are usually explained by particular sub-types being associated with more advanced disease.

Subsequent to the review by Hermanek *et al* (1995), there have been several conflicting reports on the relationship between histological variables and survival. An Estonian study of 406 patients treated by radical gastrectomy found in a multivariate analysis that in addition to stage, nodal status, extent of gastrectomy and age; papillary, tubular and undifferentiated tumours offered better survival (Arak and Kull, 1994). A study of 895 Spanish patients found a survival benefit for patients with intestinal Lauren type (Jimeno-Aranda *et al*, 1996), although this study included stage IV patients, whereas a small Swedish study of 88 patients found a benefit for diffuse Lauren type in a univariate analysis (Athlin *et al*, 1996). A population-based study of 325 patients from France reported that along with age, tumour stage, nodal status, presence of metastases, site and gross type, Ming's infiltrative type was associated with lower survival in a multivariate analysis (Roy *et al*, 1998). In a Japanese series of 195 patients who received curative resection a survival benefit was demonstrated in a multivariate model for well-differentiated tumours together with number of involved nodes and depth of invasion (Adachi *et al*, 1994). Well-differentiated tumours were also associated with improved survival in a study of 3926 patients from South Korea. They were included in a multivariate model with tumour stage, nodal status, gross type and location (Kim *et al*, 1994).

A prospective Japanese study of 647 patients (Iwasaki *et al*, 1986) found a significant difference in 5-year survival (57 *vs* 39% in advanced cases) between patients with more or less than 100 eosinophils infiltrating the tumour. In the MRC study, tumour stage, nodal status, clinical stage, WHO predominant type, Lauren predominant type, Ming type, Lauren mixed type, differentiation, tumour stroma and eosinophilic infiltration were all significant at the 5% level in the univariate analysis. However, when a multivariate survival model was chosen, only eosinophils had a significant effect once clinical stage was included in the model. Thus any apparent benefit for a particular histological subtype would appear to be explained by that subtype being associated with less advanced disease. A study based on a subset of patients entered into the Dutch gastric cancer surgical trial found on univariate analysis, that the amount of both lymphocytic and eosinophilic infiltration were of significant prognostic value (eight patients). Along with TNM stage, marked lymphocytic infiltration was also associated with better survival in multivariate analysis. However, only 105 out of 996 eligible patients were included in this analysis.

One of the difficulties of determining whether a high level of eosinophilic infiltration influences survival in gastric cancer is that the extent of eosinophil stromal infiltration has not been standardized. In the present study based on the MRC trial, as well as those described by Yu *et al* (1995) and Songun *et al* (1996), eosinophil infiltration has been graded into three groups: none or few, moderate or scanty, marked or numerous. Only Iwasaki *et al* (1986) described a more objective index: none, <100 cells, >100 cells. The subjectivity of these definitions can lead to a high level of inter-observer variation in assessments by different pathologists. This is best demonstrated by Yu's study (Yu *et al*, 1995), where eosinophils graded by one pathologist had a significant effect on survival whereas the assessments of the other review pathologist were not found to be significant. We could not validly assess the inter-observer variation in the present study as only one reference pathologist examined the majority of the pathological slides. Undoubtedly this is a weakness of the present study.

Tumour stromal eosinophilic infiltration has also been documented and investigated in a study of 38 early gastric cancers (EGC). In this study electron microscopy showed tumour stromal eosinophils with morphological evidence of activation and some tumour cells in intimate contact with activated eosinophils exhibited focal cytopathic changes (Caruso *et al*, 1993). Gastric carcinomas have been shown to express eosinophil chemotactic cytokines including IL-2, IL-5 and GM–CSF and expression of GM–CSF appears to be specific for signet ring carcinoma cells (Hong *et al*, 1999).

An early report in 1983 from the Cleveland clinic on 67 colorectal carcinomas reported great variability in the number of eosinophils in histological sections of the tumours but demonstrated a positive correlation between the numbers of stromal eosinophils and survival time (Pretlow *et al*, 1983). Two recent larger reports on stromal eosinophilic infiltration of colorectal cancer document a similar beneficial effect on prognosis (Nielsen *et al*, 1999; Fernandez-Acenero *et al*, 2000). The improved prognosis associated with the presence of marked tumour-associated tissue eosinophilia (TATE) in two cancers at either end of the gut is interesting and merits further investigation even if this is only found in a small percentage (8% in the present study) of these gastrointestinal cancers. Abnormal recruitment of tissue eosinophils is encountered in a variety of medical conditions including specific malignancies e.g., Hodgkin's disease certain types of leukaemia (Ogata *et al*, 1998) and some solid tumours (Pretlow *et al*, 1983; Iwasaki *et al*, 1986; Bethwaite *et al*, 1993; Caruso *et al*, 1993; Yu *et al*, 1995; Leighton *et al*, 1996; Songun *et al*, 1996; Ono *et al*, 1997; Geisinger *et al*, 1998; Tajima *et al*, 1998; Hong *et al*, 1999; Nielsen *et al*, 1999; Moezzi *et al*, 2000). TATE may simply be a surrogate marker of a distinctive cytokine response to an infiltrative tumour. Alternatively TATE may indicate an unusual anti-tumour immune response. Th1 and Th2 reactions involve a variety of cell types. In general, type 1 cytokines induce a strong cellular immune response whereas type 2 cytokines, predominantly a humoral response. The two systems cross regulate each other. Type 2 cytokines (IL-4 and IL-5) are known to attract eosinophilic granulocytes and for this reason, TATE may reflect a combination of strong type 2 and weak type 1 responses (van Driel *et al*, 1996). Experiments on the effector phase of tumour rejection induced by vaccination with irradiated tumour cells indicate that immunisation leads to simultaneous induction of Th1 and Th2 responses (Hung *et al*, 1998). Cytokines produced by CD4(+) T cells activate eosinophils and macrophages and these may be responsible for direct tumour cell destruction. Tumour infiltrating eosinophils may also modulate angiogenesis and desmoplastic reaction (Ono *et al*, 1997; Samoszuk, 1997). Eotaxin is the most researched C-C chemokine (Fankin *et al*, 2000), its human gene has been characterized and shown to be an early response gene of cytokine-stimulated epithelial and endothelial cells (Garcia-Zepeda *et al*, 1996). It may provide the molecular basis for eosinophil recruitment in certain tumours especially of the gastrointestinal tract.
